# Tsunami-generated magnetic fields may constrain focal mechanisms of earthquakes

**DOI:** 10.1038/srep28603

**Published:** 2016-06-29

**Authors:** Issei Kawashima, Hiroaki Toh

**Affiliations:** 1 Graduate School of Science, Kyoto University, Sakyo-ku, Kyoto 6068502, Japan; 2 Japan Manned Space Systems Corporation, Chiyoda-ku, Tokyo 1000004, Japan

## Abstract

A geomagnetic observatory named SFEMS is being operated on the deep seafloor in the northwest Pacific since August, 2001. SFEMS is capable of measuring both scalar and vector geomagnetic fields as well as the seafloor instrument’s precise attitudes, which makes it a powerful tool in detecting the so-called oceanic dynamo effect. It was found that SFEMS captured clear magnetic signals generated by the giant tsunamis of the 2011 Tohoku Earthquake even for an epicentral distance of larger than 1500 km. Here we report estimates of the focal mechanism of a closer tsunamigenic earthquake in January, 2007 on the seaward slope of the Kuril Trench using tsunami-generated variations in the observed downward magnetic component. Three-dimensional solutions of the tsunami-generated magnetic components were calculated by a new numerical code based on non-uniform thin-sheet approximation and particle motions of seawater using the linear Boussinesq approximation. As a result, a southeast dipping fault alone reproduced the dispersive nature of the downward magnetic component, while any northwest dipping faults could not. This implies that the tsunami-generated electromagnetic fields are useful for determination of focal mechanisms of tsunamigenic earthquakes, since fault dips are one of the most difficult source parameters to estimate even in modern seismology.

A large tsunamigenic earthquake occurred along the Kuril-Kamchatka trench in January, 2007 (Table 1). Its teleseismic and tsunami waveform inversions (e.g., ref. 1) have revealed that the focal mechanism was a normal fault type. However, those inversions failed to determine whether the fault dip lied in the northwest or southeast directions. The associated tsunamis were also detected^2^ by a seafloor geomagnetic observatory operating in the northwest Pacific (Fig. 1). The so-called oceanic dynamo effect was first studied by Faraday^3^. Since then, study on this phenomenon had been focused in one part on long-period oceanic waves for decades^4^. The phase velocity of such waves is typically slower than 10 m/s, and thus self-induction of the associated magnetic field is negligible. However, this doesn’t apply to tsunamis with phase velocities faster than 100 m/s and self-induction of the tsunami-generated magnetic fields^5^ should be taken into consideration as in the case of ocean tides^6^.

The advent of long-term EM observation on the seafloor^7^,^8^,^9^ enabled detection of the oceanic dynamo effect by tsunamis. As for numerical simulation of the tsunami-generated EM fields, 2-D^10^,^11^ and 3-D^12^ modelling have been proposed. Study on the tsunami-generated EM fields can contribute not only to tsunami early warning systems but also to estimation of both electrical structures beneath the ocean and focal mechanisms. Here we focus on the estimation of the focal mechanism of the East of Kuril Islands Earthquake in January, 2007 using the observed tsunami-generated EM data.

Our SeaFloor ElectroMagnetic Station (SFEMS) installed at a site in the Northwest Pacific Basin (NWP) recorded scalar and vector (black dots in Fig. 2) components of the geomagnetic field every two minutes when the tsunamigenic earthquake occurred. The scalar and vector measurements of the geomagnetic field were conducted by an Overhauser proton precession magnetometer and a three-component fluxgate-type variograph with resolutions of 0.1 nT and 0.01 nT, respectively. SFEMS is also equipped with a fibre optical gyro, a two-component horizontal tilt-meter with a resolution of 0.9 arcsec and a thermometer with a resolution of 0.01 °C for post-retrieval calibration. It was geomagnetically very quiet when the tsunami arrived at the seafloor observatory on January 13, 2007 (Fig. S1 of Supplementary Information) and thus no correction for the external geomagnetic fluctuations was necessary.

We searched for the slip distribution that best explained the observed vector magnetic data. We assumed that the observed field consisted of linear superposition of magnetic fields arising from each sub-fault. Using the dislocation theory for a rectangular fault model, the linear Boussinesq equations (see Eqs (4) through (6) in Methods Section) for tsunami propagation were solved in addition to the Fredholm integral equation of the 2^nd^ kind for EM simulation (Eqs (11) and (12)) based on the non-uniform thin-sheet approximation (Eq. (9)). The amount of slips for each sub-fault was obtained by the non-negative least squares method^13^. Table 2 shows the slip distributions of Fault Models A and B. As for the locations and geometry of each sub-fault, refer to Fig. S2 and Table S1 in Supplementary Information. Figures 2a through 2d show the comparison of the observed and calculated downward magnetic components, two horizontal magnetic components, and the calculated surface elevation at the location of the EM observatory, respectively. The variances of the model fits for the observed downward magnetic field were 3.89 for Fault Model A and 2.12 for Fault Model B. This indicates that Fault Model B reproduced the downward magnetic component better than Fault Model A. However, the variance ratio was 1.83, which was slightly smaller than the critical *F*-value of 1.84 corresponding to 95% confidence level, although it passed the 90% *F*-test. Fault Model B also gives a better fit to the eastward magnetic component than Fault Model A, though the fits to the northward component are marginal.

The major difference between Fault Model A and B is their ability in generating high-frequency magnetic fluctuations after the first arrival of the tsunami, which originally stems from the dispersive nature of the 2007 Kuril tsunami (see Fig. S3). The better fits of Fault Model B predictions to the observed vector magnetic data clearly show that the southeast dipping fault is capable of generating the dispersive tsunami at the site of SFEMS, while the northwest dipping fault is not. However, the limited time resolution of our data (2 minutes) prevents us from further argument on tsunami dispersion. Although Fault Model B predicts high frequency tsunami phases and gives a better fit, the vector magnetic data at the seafloor could not resolve them completely especially for periods 70 minutes after the origin time of the tsunamigenic earthquake. This implies that higher time resolution is necessary for future seafloor EM instruments targeting tsunami studies. The sampling rate should be shorter than 60 s and preferably variable on demand from land stations with ‘satellite’ as well as ‘acoustic’ link to the seafloor^14^.

It is also noteworthy that the small variations of the northward magnetic component approximately 52 min after the earthquake’s origin time can be interpreted as ‘initial rises^10^’, which is a necessary result from the counter electric field induced in front of the tsunami due to self-induction within the conductive seawater. The surface elevation of Fault Model B, i.e., the southeast dipping model, explains the observed surface elevation better than Fault Model A at DART 21413 and 21414.

In conclusion, the EM signals generated by the tsunami at the time of the Kuril earthquake on January 13, 2007 were detected successfully by our seafloor geomagnetic observatory with the clear frequency dispersion effect. A new 3-D simulation code for tsunami-generated EM fields in frequency domain was developed, which included not only the inducing non-uniform source fields generated by particle motions of conductive seawater but also self-induction within the ocean and its substrata, in order to determine the fault dip of the source earthquake that emitted the dispersive tsunami. We conducted kinetic and EM simulations based on three previously reported fault models to find none of them compatible with the observed downward magnetic component. The slip distribution on sub-faults was then optimised to yield the best-fit southwest-dipping fault model. This means that the EM observatories like SFEMS can contribute to determine focal mechanisms more precisely than using seismic stations alone.

## Methods

### Numerical method for tsunami simulation

For kinetic simulation of tsunami propagation, we solved the linear Boussinesq momentum equation by modifying the following finite-difference code: Cornell Multi-grid Coupled Tsunami Model (COMCOT^15^, Version 1.7). COMCOT originally employed the linear shallow water momentum equation in the spherical coordinate system and uses an explicit leapfrog finite-difference method for its time evolution. The linear shallow water momentum equations can be given as follows;


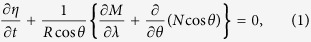



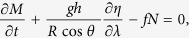



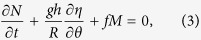


where *η* and *h* denote the sea surface elevation and the still water depth, respectively. (*M*, *N*) represent the volume flux, defined by products of velocity and water depth, in the west-east and south-north directions each. (*θ*, *λ*) are respectively the latitude and longitude, and *R* is the mean radius of the Earth while *g* is the constant gravitational acceleration on the Earth’s surface. *f* denotes the Coriolis force coefficient and is equal to *Ω* sin *θ* with *Ω* for the rotation rate of the Earth.

The dispersion effect on the tsunami propagation in the deep ocean should be taken into account even for near field tsunamis. We, therefore, modified COMCOT version 1.7 to accommodate the linear Boussinesq momentum equations, which can be written as follows:


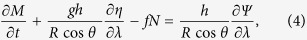



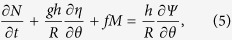



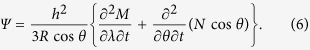


The right hand terms of Eqs (4) and (5) are the source of the dispersion effect. The following Poisson’s equation can be obtained from Eqs (1) and (4) through Eq. (6):


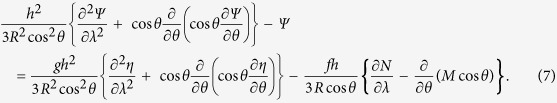


The kinetic simulation process can be separated into three steps: We first solved the continuum equation (1) by a leapfrog finite-difference method. Then, we solved the linear shallow water momentum equations (2) and (3) on the lateral boundaries to obtain boundary values of 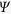 using Eq. (6). We successively solved the Poisson’s equation (7) by an implicit method using LU decomposition. Finally, we solved the linear Boussinesq momentum equations of (4) and (5). An example of prominent dispersion reproduced by the modified COMCOT is given in Fig. S3 of Supplementary Information.

### Evaluation of tsunami-generated 3-D EM fields

The induction equation for the magnetic field, ***b***, in the frequency domain is given by;


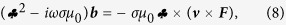


for a uniform conductivity, σ. Here, ω and μ _0_ are the angular frequency and the magnetic permeability in vacuum, respectively while ***v*** and ***F*** are the velocity of the moving conductor and the ambient magnetic field (|***b***| ≪ |***F***|) each.

We used the following non-uniform thin-sheet approximation^16^ in order to solve Eq. (8) for an angular frequency ω;


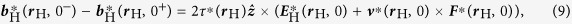


where quantities with the superscript * and the subscript _H_ denote non-dimensional and horizontal vectors, respectively. We scaled the magnetic field ***b***in units of vertical component of the geomagnetic main field (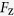), all lengths in units of the skin depth in the first layer beneath the surface (*z* = 0) thin sheet (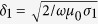) and thus the velocity was measured in units of 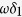. The electric field ***E*** was given in units of 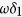

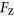
and the conductance τ within the thin sheet in units of 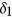

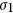
. The remaining two vectors, ***r*** and 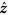, are a position vector and a unit vector in the downward direction, respectively. Equation (9) means that the gap in the horizontal magnetic components above (0^−^) and below (0^+^) the thin sheet is equal to the net electric current flowing within the thin sheet.

Assuming the substrata beneath the top thin sheet are horizontally stratified and imposing the following boundary conditions at lateral infinities;


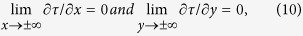


the induction equation for the horizontal electric field within the thin sheet is reduced to the following Fredholm integral equation of the 2^nd^ kind;


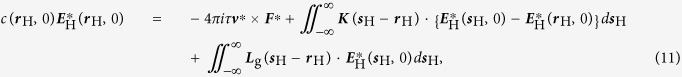


where ***K*** and ***L***_g_ are the Green’s tensors^16^,^17^ and 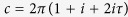. Using the horizontal electric field within the thin sheet, E* thus derived, the horizontal components of the tsunami-generated magnetic field on the seafloor is given by;


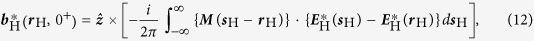


where ***M*** is the Green’s tensor and function^17^ again. The vertical magnetic component can be calculated by taking curl of E*.

## Additional Information

**How to cite this article**: Kawashima, I. and Toh, H. Tsunami-generated magnetic fields may constrain focal mechanisms of earthquakes. *Sci. Rep.* **6**, 28603; doi: 10.1038/srep28603 (2016).

## Supplementary Material

Supplementary Information

## Figures and Tables

**Figure 1:**
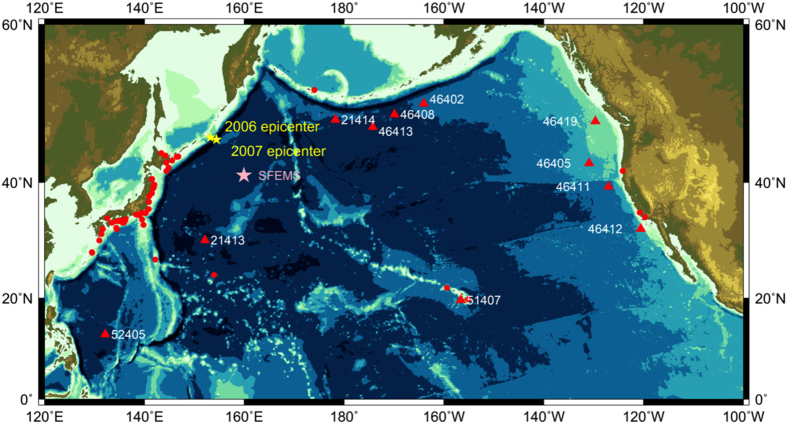
Site map of the north Pacific Ocean. Epicenters of the November 2006 and the January 2007 Kuril earthquakes (yellow stars). Red circles and triangles indicate the location of available tide stations and DART buoys, respectively. A pink star indicates the location of our seafloor electromagnetic station (SFEMS). This figure was created using Generic Mapping Tools (GMT)^18^ v4.5.14 available at http://www.soest.hawaii.edu/gmt/.

**Figure 2:**
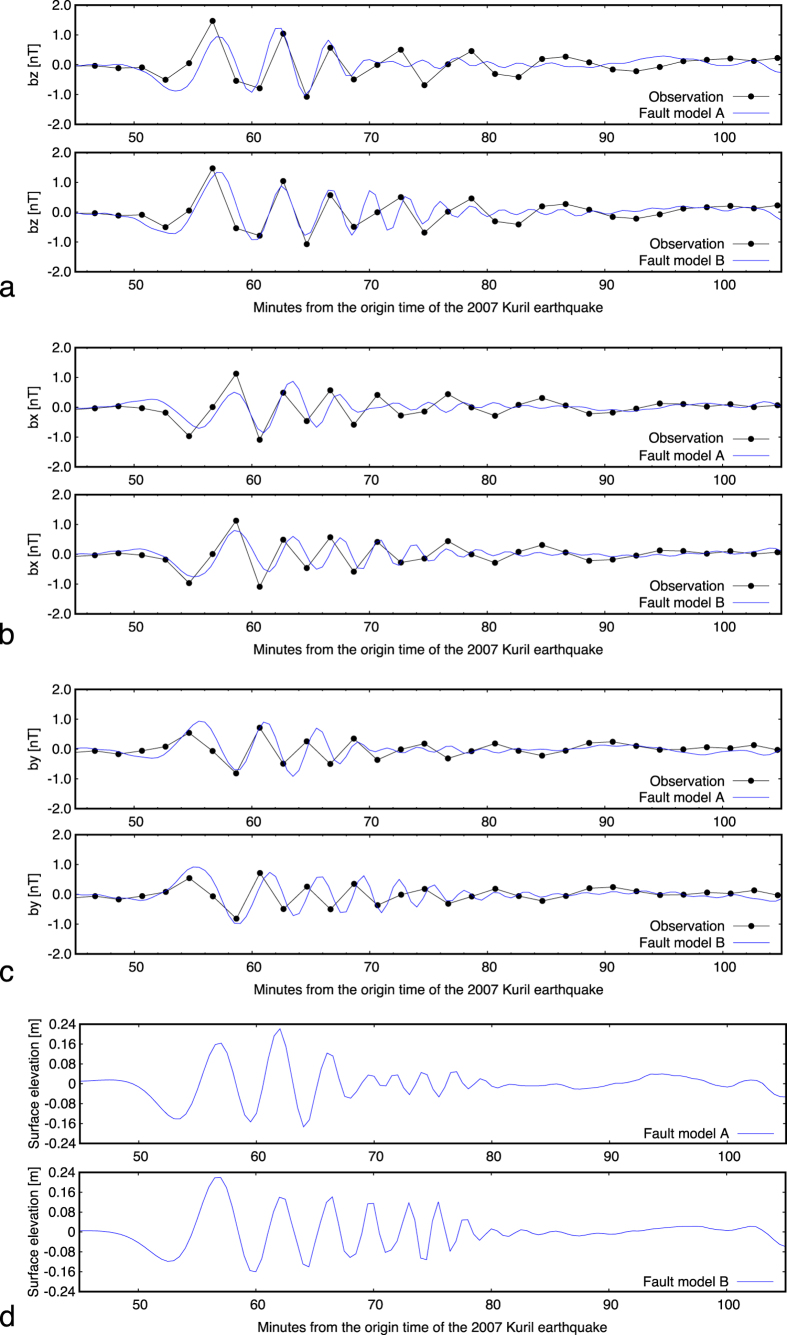
Observed vector magnetic data and two fault model prediction. (**a**) Downward magnetic component (*b*_z_). Black dots are the observed data. Blue curves in each panel show the predictions by the best Fault Models A and B. (**b**) Same as (**a**) but for northward magnetic component (*b*_x_). (**c**) Same as (**a**) but for eastward magnetic component (*b*_y_). (**d**) Calculated surface elevations for both fault models at the EM observatory.

**Table 1:** Earthquake and site descriptions.

Latitude [°N]	Longitude [°E]	Depth [km]	Origin Time	Moment Magnitude
			Epicentral Distance [km]	Site Name
46.272	154.455	10.0	January 13, 2007 04:23:20 UTC	8.1*
41.102	159.963	5.58	725.7	SFEMS^2^

^*^Details of the tsunamigenic earthquake can be obtained from United States Geological Survey (USGS) at http://earthquake.usgs.gov/earthquakes/eqinthenews/2007/us2007xmae/.

**Table 2:** Slip distribution on each subfault.

Subfault Number	1	2	3	4	5	6
Fault Model A [m]	3.64	9.96	0.700	0.000	0.000	7.41
Fault Model B [m]	4.19	11.0	0.000	0.000	0.000	1.37
