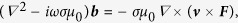# Erratum: Tsunami-generated magnetic fields may constrain focal mechanisms of earthquakes

**DOI:** 10.1038/srep31260

**Published:** 2016-08-25

**Authors:** Issei Kawashima, Hiroaki Toh

Scientific Reports 6: Article number: 28603; 10.1038/srep28603published online: 06 29 2016; updated: 08 25 2016

In the HTML version of this Article, there are typographical errors in Equation 8,
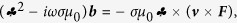


should read: