# Supplementary material for: Tsunami-generated magnetic fields may constrain focal mechanisms of earthquakes
Source: Sci Rep. 2016 Jun 29;6:28603. doi: 10.1038/srep28603 (PMC4934024; doi:10.1038/srep28603)
Supplement: Supplementary Information [file srep28603-s1.pdf]

Tsunami-generated magnetic fields may constrain focal mechanisms of earthquakes

Issei Kawashima[1, 2] & Hiroaki Toh[1]

[1] *Graduate School of Science, Kyoto University, Sakyo-ku, Kyoto 6068502, JAPAN*

[2] *Japan Manned Space Systems Corporation, Chiyoda-ku, Tokyo 1000004, JAPAN*

Effect of geomagnetic disturbances of external origin

It is essential for successful detection of tsunamis by EM sensors that the tsunami-generated EM fields should be identified clearly from other sources of geomagnetic temporal variations. The most prominent noise source here is geomagnetic disturbances of external origin such as those created in the magnetosphere and/or the ionosphere.

The K_p index is known to a measure of external geomagnetic activities and is evaluated every three hours at Potsdam in Germany using data from 13 geomagnetic observatories worldwide. ΣK_p^* is a daily sum of the 8 K_p indices representing the strength of the geomagnetic activity on that day.

It is evident from Fig. S1 that it was geomagnetically very quiet when the tsunamigenic earthquake occurred on January 13, 2007. That day is actually classified as the quietest day of the month (Q1). It, therefore, is unlikely that the original EM time-series observed on the seafloor are contaminated by temporal geomagnetic variations of external origin.

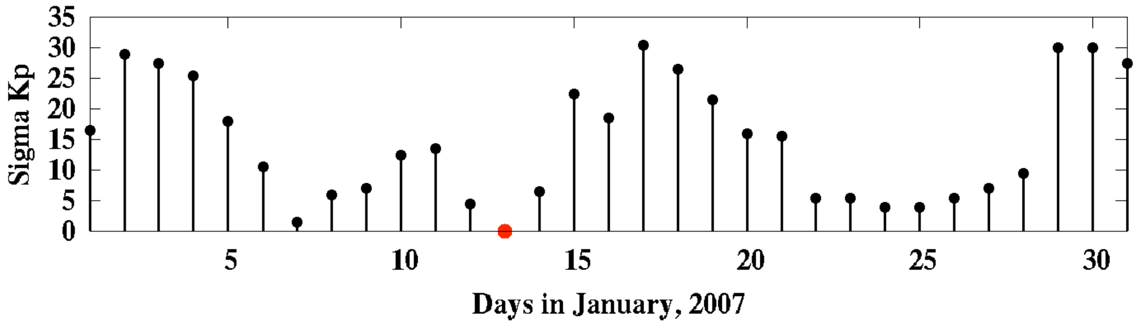

Fig. S1 The daily sums of K_p indices in January, 2007. The red dot indicates the ΣK_p index on January13, 2007.

* ΣK_p is calculated at Helmholtz-Zentrum, GeoForschungsZentrum, Potsdam in Germany and can be downloaded from:

http://www.gfz-potsdam.de/en/section/earths-magnetic-field/services/kp-index/

Fault geometry of the Kuril tsunamigenic earthquake in 2007

The geometry of the two candidate faults in Table 2 of the main text is described here.

As a representative of north-west dipping faults, we selected Fault Plane A* (see Fig. S2) among the existing source mechanisms for the Kuril earthquake in 2007. This fault plane was determined by teleseismic body wave inversion. For that of south-east dipping faults, Fault Plane B# also in Fig. S2 was chosen and is a global CMT solution. We assigned six square subfaults with a length and width of 40km based on Fujii and Satake's[A1] subfault setting for each fault plane. Deformation of the seafloor was calculated using the dislocation theory[A2] for a rectangular fault model and the effect of horizontal displacement was also considered[A3]. We assumed that the deformation lasted for 50s at a constant speed on each subfault.

We also tried Fault Plane C$ in examination of strike dependence to find this geometry always yielded no better fits to the data than Fault Plane B and thus was not considered any further. All fault parameters are given in Table S1.

* Details of the fault plane can be found on the Univ. Tsukuba website:
http://www.geo.tsukuba.ac.jp/press_HP/yagi/EQ/2007Chishima/

This plane is given at http://www.globalcmt.org.

$ Fault Plane C is described in:
http://earthquake.usgs.gov/eqcenter/eqinthenews/2007/us2007xmae/finite_fault.php

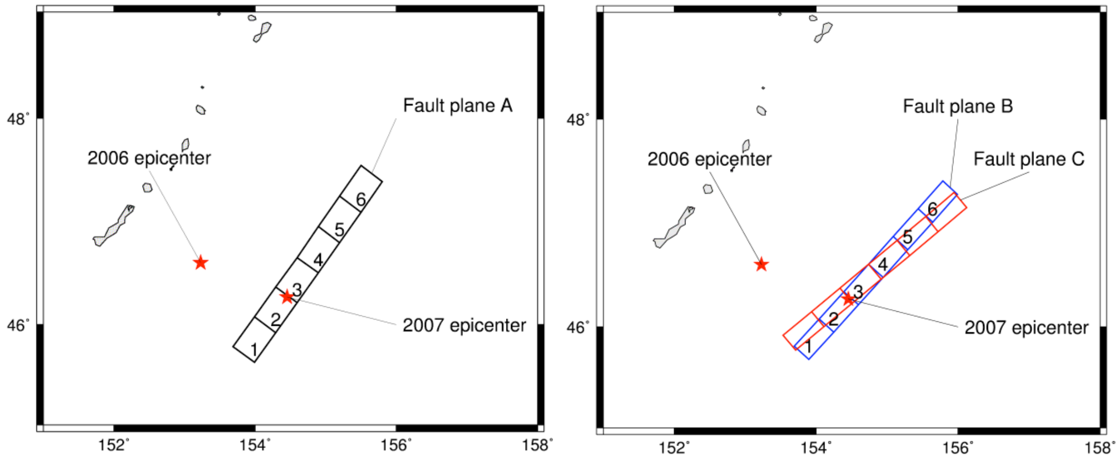

Fig. S2 (Left) Location of the northwest-dipping Fault Plane A. (Right) Those of the southeast-dipping faults: Fault Plane B (blue rectangles) and Fault Plane C (red rectangles). Each subfault is numbered from southwest to northeast. These

figures were created using Generic Mapping Tools (GMT) [18] v4.5.6 available at http://www.soest.hawaii.edu/gmt/.

Table S1: Attitude parameters of each fault plane.

	Strike	Dip	Rake
Fault Plane A	N215°E	45°	-110°
Fault Plane B	N42°E	58°	-114°
Fault Plane C	N50°E	58°	-114°

Dispersive nature of the Kuril tsunami event in 2007

In order to demonstrate the ability of the modified COMCOT code in reproducing the Kuril tsunami's dispersion, here we show a snapshot of surface elevation at a certain elapsed time in Fig. S3. It is evident from the figure that our seafloor EM observatory is located in a region of prominent dispersive effects, i.e., in the midst of the red and blue stripes at that moment, which has never seen in the case of the linear long-wave approximation (the right diagram of Fig. S3). The linear Boussinesq approximation alone reproduced the dipsersion of the tsunami in concern successfully.

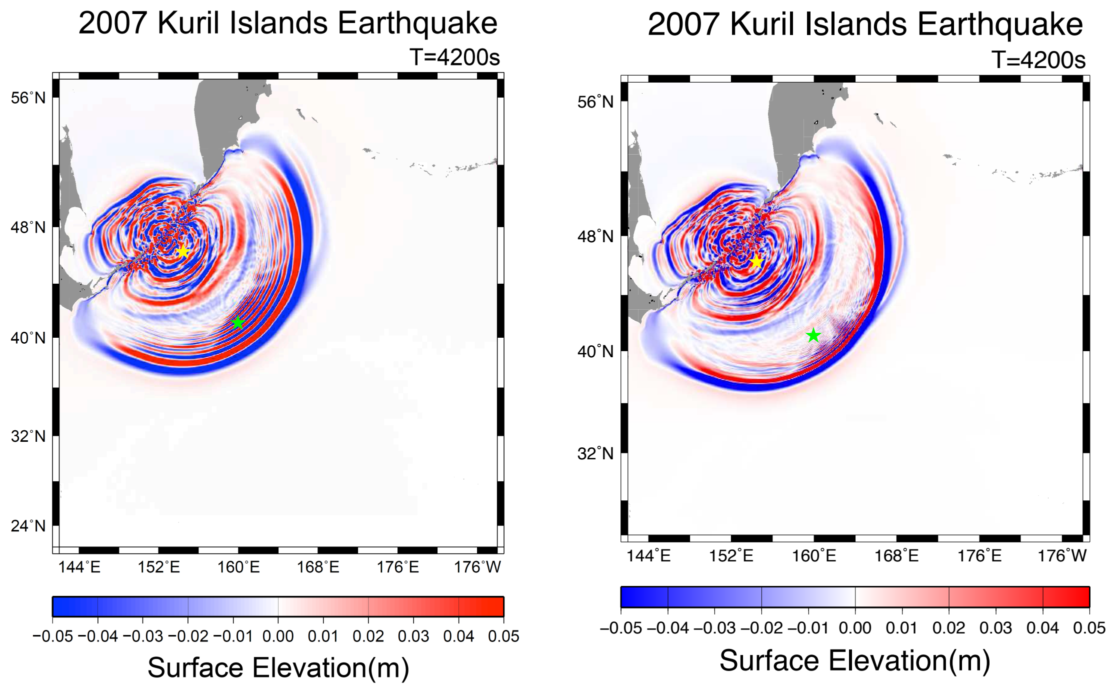

Fig. S3 Surface height estimates by the linear Boussinesq approximation (left) and by the linear long-wave approximation (right) at an elapsed time of 4200s from the earthquake's origin time. The yellow stars indicate the epicenter of the January 2007 Kuril earthquake. The other green stars denote the location of the EM observatory. It is evident that the tsunami dispersion clearly manifests at the observatory location in the case of the linear Boussinesq approximation. These figures were created using Generic Mapping Tools (GMT) [18] v4.5.6 available at http://www.soest.hawaii.edu/gmt/.

Additional References

A1. Fujii, Y. & Satake, K. Tsunami sources of the November 2006 and January 2007 great Kuril earthquakes. *Bull. Seismol. Soc. Am.* **50**, 1559-1571 (2008).

A2. Okada, Y. Surface deformation due to shear and tensile faults in a half-space. *Bull. Seismol. Soc. Am*. **75**, 1135-1154 (1985).

A3. Tanioka, Y. & Satake, K. Tsunami generation by horizontal displacement of ocean bottom. *Geophys. Res. Lett*. **23**, 861–864 (1996).
